# Identification of gene targets regulated by the IclR-like regulator SL1344_3500 in *Salmonella* Typhimurium

**DOI:** 10.1128/jb.00054-25

**Published:** 2025-08-25

**Authors:** Thea B. Andersen, Nicolas Näpflin, Noemi Santamaria de Souza, Christopher Schubert, Bidong D. Nguyen

**Affiliations:** 1 Department of Biology, Institute of Microbiology, ETH Zurich 30845 , Zurich, Switzerland; 2 Department of Molecular Life Science, University of Zurich 400018, Zurich, Switzerland; University of Virginia School of Medicine, Charlottesville, Virginia, USA

**Keywords:** *Salmonella*, microbioal pathogenesis, microbiota, metabolism, gene regulation, xylonate, sugar acids, inflammation

## Abstract

Transcriptional regulation of metabolic operons is important for optimal carbohydrate use and for mitigating the accumulation of toxic intermediates. Here, we characterize *SL1344_3500*, encoding a putative IclR-like regulator in *Salmonella enterica* Typhimurium. We present genetic and transcriptional evidence that it regulates the expression of two neighboring operons, one designated here as *xynABC*, enables utilization of xylonate as a sole carbon source. Furthermore, our findings indicate that *SL1344_3500* is important for luminal growth in several mouse models, exerting its effects through the suppression of the *xynABC* operon. Based on the observation that the Δ*SL1344_3500* deletion can be stably complemented *in vivo*, we developed a plasmid stabilization strategy. This gene complementation approach shows promise for generating stable gene reporters for long-term colonization experiments.

**IMPORTANCE:**

Understanding transcriptional regulation in *Salmonella enterica* Typhimurium is crucial for revealing how enteric pathogens optimize metabolism to compete with commensals in the gut. SL1344_3500, an IclR-like transcriptional regulator controlling genes linked to sugar acid metabolism, is essential for luminal growth in mouse models through gene suppression and represents a potential target for antimicrobial development. Based on these observations, we developed stable reporter plasmids that use gene complementation of SL1344_3500 to prevent plasmid loss during long-term *in vivo* studies.

## INTRODUCTION

Transcriptional regulation is a key component of bacterial physiology, enabling adaptation to an ever-changing environment. Transcriptional regulators can affect expression of a target gene in response to external stimuli. One such class of regulatory proteins is the isocitrate lyase (IclR)-like regulators, which are abundantly found in gram-negative and gram-positive bacteria, as well as in Archaea, and are involved in diverse functions beyond isocitrate metabolism (1). These functions include sugar acid and aromatic amino acid metabolism, and virulence-associated traits, such as biofilm formation and multidrug resistance (1). These regulators can both be repressors or activators, and interestingly, their activity is modulated by binding of effector molecules that can either induce or repress the expression of the target genes (1).

The enteric pathogen *Salmonella* Typhimurium (*S*. Tm) SL1344 contains six IclR-like regulators, and with the exemption of *SL1344_3500*, all share high sequence identity (>85%) to the orthologous *Escherichia coli* genes (1, 2). Although IclR-like regulators in *S*. Tm remain poorly characterized, their function can be inferred from studies of *E. coli*, a close relative with highly similar gene sequences. The two organisms differ in ecology and pathogenicity, however, leading to species-specific differences in these regulators, and certain features of the IclR-like regulators and their regulons have been directly linked to *Salmonella* virulence (3–7).

*SL1344_3500*, which encodes for putative IclR-like regulator, was identified as a hit in a transposon mutagenesis screen and was found to significantly contribute to growth in the murine gut. Mutations in *SL1344_3500* confer a severe fitness defect in colonizing a mouse model with an intact gnotobiotic microbiota (8). In this work, we characterized *SL1344_3500* and assessed how it contributes to *Salmonella* fitness in the gut using several murine infection models. We employed computational and genetic tools to examine the potential target genes of this regulator and the pathway that they are involved in. Additionally, we utilized the fitness attenuation of the Δ*SL1344_3500* strain as a molecular tool to ensure reliable plasmid maintenance. Since a plasmid copy of this gene can stably complement the growth attenuation of the regulator-deficient mutant, we applied this observation to enhance plasmid stabilization for gene reporters. This gene complementation strategy can be useful for generating stable gene reporters that can be maintained without the need for antibiotics, enabling long-term analysis of pathogen gene expression in an unperturbed microbiota.

## RESULTS

### Comparative analysis of SL1344_3500

Based on sequence homology, the *SL1344_3500* open reading frame (ORF) of *S*. Tm SL1344 is predicted to encode an uncharacterized transcriptional regulator belonging to the family of IclR-like regulators (9). AlphaFold prediction models reveal that *SL1344_3500* encodes a 251-amino acid protein containing a characteristic DNA binding N-terminal winged helix-turn-helix domain found specifically in this family of regulators, as well as a C-terminal effector binding domain that typically binds small regulatory substrates (10). Based on these analyzes, we can assume with high confidence that *SL1344_3500* encodes a transcriptional regulator of the IclR-like family that alters gene expression in response to yet an unknown effector molecule.

**Fig 1:**
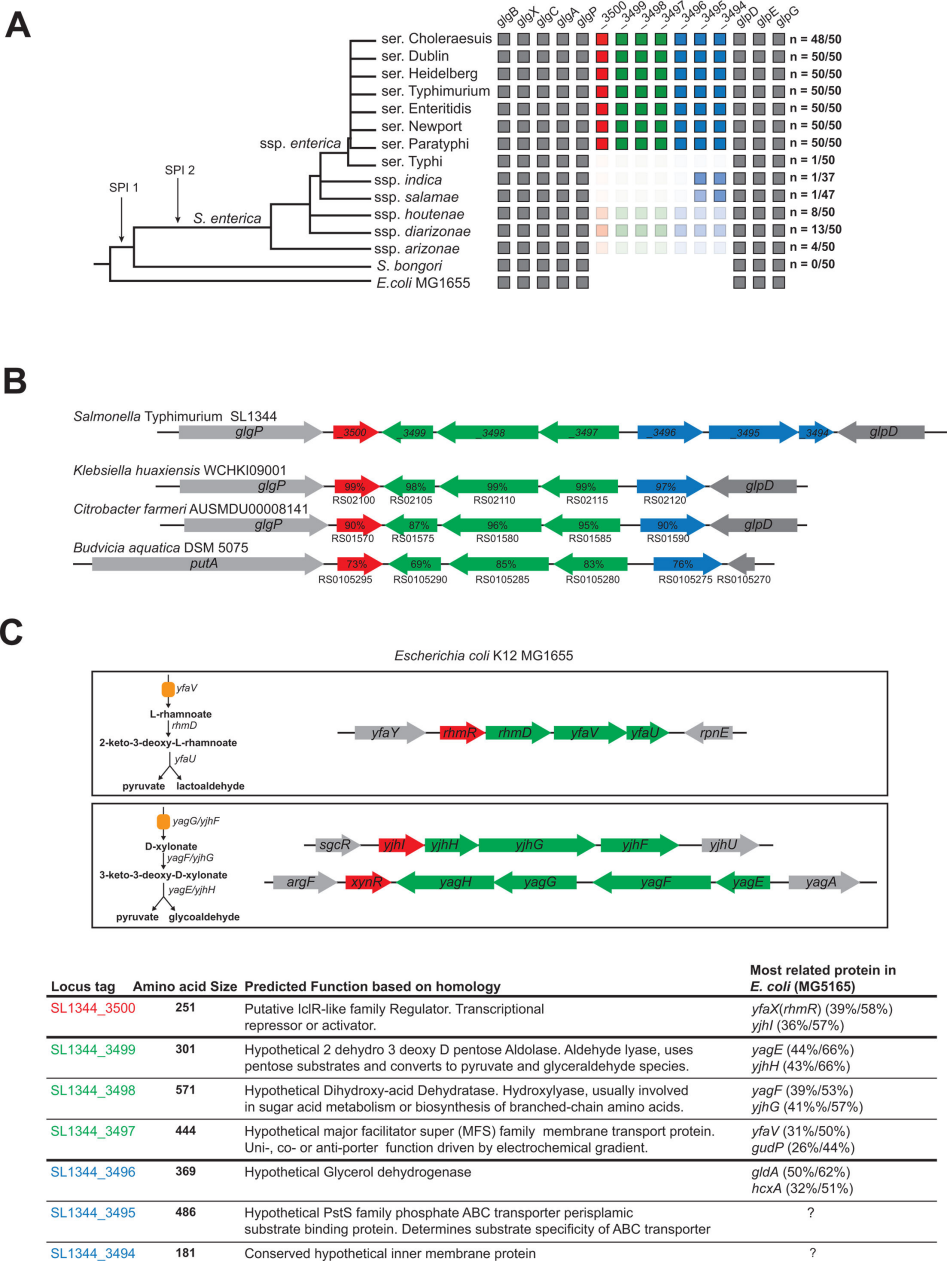
Homologous versions of SL1344_3500 and their neighboring operons have a pattern of co-occurrence and are found in Clade A of *S. enterica* subsp. *enterica*. (**A**) Genomes (*n* ≤ 50, when available) were randomly selected for the indicated subspecies or serovars. Boxes represent the presence of a gene, while the color intensity reflects the proportion of strains within each serovar or subspecies that carry the gene. The number indicates the number of genomes for which SL1344_5000 and both operons are found. Phylogenetic tree modified from Desai et al. (11). (**B**) Examples of *SL1344_3494-SL1344_3500* homologs identified in other Enterobacteriaceae members. Percent amino acid identity is shown. Wherever a gene annotation is missing, sequence locus tags are provided. (**C**) Predicted functions based on homology from reference *E. coli* MG1655 genome. Percent amino acid identity and similarity in parentheses.

Reciprocal best hit searches revealed that SL1344_3500 homologs are found in other *Salmonella* spp. (Fig. 1). Bioinformatics analysis of randomly selected *Salmonella* genomes from the major serovars of *S. enterica* subsp. *enterica*, as well as other *Salmonella* subspecies, revealed that SL1344_3500 homologs were present in most nontyphoidal members of Clade A and in most serovar Paratyphi strains while entirely absent in serovar Typhi, except for one strain (Fig. 1A). These SL1344_3500 homologs were sparsely represented in other subspecies and were absent in *Salmonella bongori*. The absence of these genes may suggest that their loss is positively selected during host specialization (Fig. 1A). Interestingly, homologs of SL1344_3500 consistently co-occur with homologs of SL1344_3494-3499 at the same genomic locus.

SL1344_3500 homologs and associated genes were also identified in other members of the Enterobacteriaceae family. In our analysis, we found that SL1344_3500 homologs are, however, only sparsely detected across diverse Enterobacteriaceae lineages, including *Atlantibacter*, *Citrobacter*, *Klebsiella*, *Budvicia*, *Limnobaculum*, and *Leminorella* (Fig. 1B; Fig. S1). When SL1344_3500 homologs were identified, they typically appeared in only one to a few strains within a given genus. For example, we found the SL1344_3500 homolog in four *Klebsiella* species (*K. huaxiensis*, *K. spallanzani*, *K. electrica*, and unclassified *Klebsiella* species) but not in other analyzed members of the *Klebsiella* genus (24 representative *Klebsiella* genomes) (Fig. S1). Similarly, only three out of 26 analyzed *Citrobacter* species (*C. farmeri*, *C. telavivensis*, and *C. amalonaticus*) were found to contain the SL1344_3500 homolog. Notably, *K. huaxiensis* strain WCHKI09001, *C. farmeri* AUSMDU0008141, *C. telavivensis*, *C. amalonaticus* and *Budvicia aquatica* DSM 5075, as well as *Atlantibacter* species, carry not only SL1344_3500 homologs but also homologs of the neighboring operons, SL1344_3496-3499 (Fig. 1B; Fig. S1). However, homologs of SL1344_3494 and SL1344_3495 were absent in these genomes. Given the high amino acid sequence identity (69% to 99%), as well as the conserved gene order, these homologs are likely to share a recent common ancestry. In *K. huaxiensis* and the *Citrobacter* species, the SL1344_3500 homolog and associated gene clusters were located in the same genomic region, between *glgP* and *glpD*, as in *S*. Tm SL1344 (Fig. 1B). In *B. aquatica*, however, while the homologs were not found exactly at the same genomic location, they were positioned nearby. Likewise, *Atlantibacter* homologs of SL1344_3500 and the neighboring operons, SL3496-3499, were located in a different genomic context (Fig. S1). Overall, these data indicate that the putative IclR-like regulator and associated operons co-occur and may have entered the genome together as a functional unit. Therefore, we hypothesize that SL1344_3500 regulates the expression of *SL344_3494-SL344_3499*.

A comparison of the amino acid sequences of the encoded genes with the annotated homologs in the model *E. coli* MG1655 provided hints about the function of these genes. The *SL1344_3500* ORF shares significant sequence similarity with YfaX (RhmR) and YjhI, which are IclR-like regulators involved in sugar acid catabolism, namely, rhamnoate and xylonate, respectively (Fig. 1C) (12–16). The regulons of both YfaX and YjhI encode functionally similar enzymes that share a degree of homology with each other, as well as with the putative products of the SL1344_3500 regulon. These operons include genes encoding for a transporter, an aldolase and dehydratase, all required for transport and breakdown of these sugar acids to pyruvate and an aldehyde. Likewise, *SL1344_3499* is predicted to encode a dihydrodipicolinate synthase (an aldolase), *SL1344_3498* a dihydroxy-acid dehydratase, and *SL1344_3497* a major facilitator superfamily transporter (Fig. 1C) (9). *E. coli* MG1655 possesses a second xylonate utilization operon regulated by an IclR-like regulator, XynR (17). This operon includes two genes, *yagE* (encoding a xylonate aldolase) and *yagF* (encoding a dehydratase), which share homology with *SL1344_3499* and *SL1344_3498*, respectively (17–20). *SL1344_3496* encodes a putative glycerol dehydrogenase, though its connection to sugar acid metabolism cannot be determined from our analysis (Fig. 1C) (9). Based on this analysis, we predict that *SL1344_3497* to *SL1344_3499* encode a xylonic acid utilization operon. This is supported by the observation that deletion of this region abrogates growth on D-xylonate as a sole carbon source (Fig. S2). The functions of the other genes remain more enigmatic, with no close homologs in *E. coli* MG1655. Based on limited homology, these genes appear to encode remnants of a transporter complex that are missing key components. *SL1344_3495* purportedly encodes a periplasmic substrate-binding protein with some homology to the high-affinity phosphate-binding protein PstS, while *SL1344_3494* encodes a putative inner membrane protein, though its function is currently unknown (Fig. 1C) (9). Based on these observations, SL1344_3500 regulates the catabolism of xylonic acid, along with additional genes of unknown function.

### SL1344_3500 inactivation alleviates repression of neighboring operons

**Fig 2:**
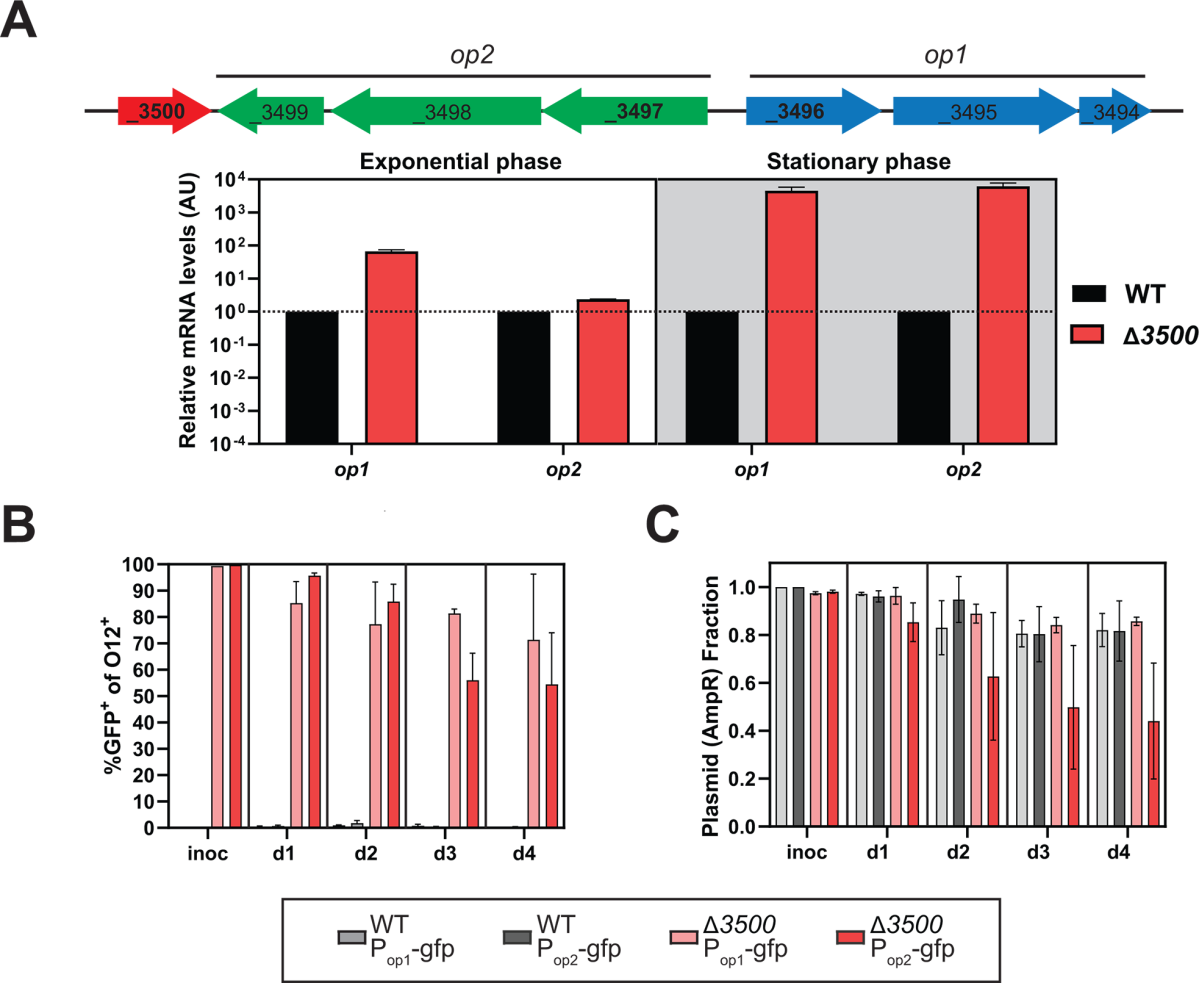
Deletion of *SL1344_3500* increases expression from the neighboring operons *in vitro* and *in vivo*. (**A**) Relative gene expression of indicated genes and strains, during exponential growth (left) and stationary phase (right). The indicated strains (wild type, WT; Δ*SL1344_3500* mutant, Δ*3500*) were grown aerobically in LB with streptomycin at 37°C with agitation. Samples were collected during exponential (OD_600_ ~0.3) and stationary (OD_600_ ~2.5) phase. The Livak (ΔΔCt) method was used to calculate relative mRNA expression levels, with *rpoD* as the endogenous reference gene and the WT strain serving as the calibrator for each condition. The experiment was performed in parallel biological duplicates and analyzed in technical triplicates. (**B**) Transcriptional GFP reporters for *op1* and *op2* in WT and the Δ*SL1344_3500* mutant (Δ*3500*). The indicated strains were used to mono-infect by oral gavage streptomycin-pretreated C57BL/6 SPF mice with ~5×10^7^ CFU. Fecal samples were subjected to immunostaining for *S*. Tm (with anti O12 antibodies) and high-throughput fluorescence analysis using flow cytometry. Samples from mice infection with a GFP^−^ and GFP^+^ control were used to set the GFP gate (not shown). (**C**) Plasmid retention as determined by replica plating of the original MacConkey CFU enumeration plate on plates with or without ampicillin.

We predict that SL1344_3500 regulates the two neighboring operons, *SL1344_3494-3496* (designated “*op1*”) and *SL1344_3497-3499* (designated “*op2*”). To test whether *SL1344_3500* controls expression of one or both of these operons, reverse transcriptase quantitative PCR (RT-qPCR) was performed on cDNA synthesized from RNA from exponential and stationary bacterial cultures. RT-qPCR primers were designed to amplify a short fragment of the first gene in the two operons. In exponential phase, higher mRNA levels of *op1* and *op2* were observed in Δ*SL1344*_*3500* compared to wild type (WT) (Fig. 2A). In stationary phase, expression of both *op1* and *op2* is greatly increased in Δ*SL1344*_*3500*, inferred from relative mRNA levels. These data suggest that SL1344_3500 negatively regulates the expression of *SL1344_3494 to SL1344_3499* and that their expression is also regulated by factors related to growth phase.

To determine the *in vivo* expression levels of *SL1344_3500* and the two predicted operons, we constructed transcriptional reporters by cloning DNA fragments upstream from each operon into green fluorescent protein (GFP) reporter plasmids. We tested these transcriptional reporters in the WT strain and in Δ*SL1344_3500* isogenic mutant in streptomycin-pretreated C57BL/6J specific pathogen-free (SPF) mice by oral gavage. GFP expression in bacteria harvested from fecal contents, fixed and stained for *S*. Tm using O12 antiserum, was analyzed by flow cytometry. Very few cells with appreciable GFP expression under *op1* and *op2* promoters were observed in the WT background compared to 50%–95% positive cells in the Δ*SL1344_3500* background (compare WT P_op1_-gfp vs Δ*3500* P_op1_-gfp and WT P_op2_-gfp vs Δ*3500* P_op2_-gfp in Fig. 2B). We tested a GFP reporter for the promoter of SL*1344_3500* but were unable to obtain reliable detection, likely due to low basal expression of this gene (data not shown). However, based on the *op1* and *op2* GFP reporters in the WT vs Δ*SL1344_3500* backgrounds, we have evidence that *SL1344_3500* is expressed *in vivo* (Fig. 2B). These observations suggest that the expression of *op1* and *op2* is negatively correlated to the presence of SL1344_3500. We observed an overall decrease in the number of GFP-positive cells in the Δ*SL1344_3500* background over the course of the infection, attributed to plasmid loss (Fig. 2C). High GFP expression has been reported to suppress *S*. Tm growth, likely due to the metabolic burden or toxic levels of expression (21). Plasmid loss may result from the negative selection pressure associated with high GFP expression.

### SL1344_3500 deficiency is detrimental in gut-luminal growth

**Fig 3:**
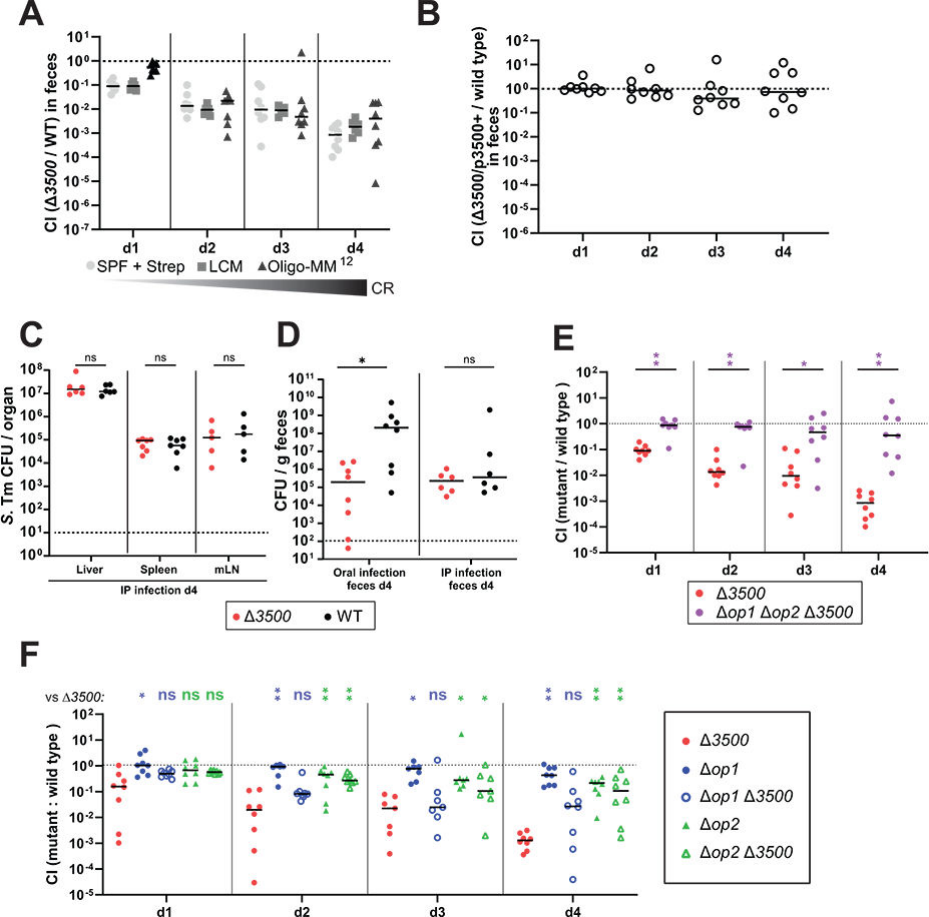
The Δ*SL1344_3500* mutant is attenuated *in vivo* irrespective of tested microbiota models. (**A**) Δ*SL1344*_*3500* mutant (Δ*3500*), wild type (WT), was tested in streptomycin-pretreated SPF mice, LCM mice, and Oligo mice. The mouse models were all derived from the C57BL/6J genetic background but differ in microbiota complexity and colonization resistance (CR), as indicated. (**B**) The complemented strain (Δ*3500*/p3500^+^; with ectopic expression of SL1344_3500 on a plasmid vector) was tested against a WT strain in oral infection model in streptomycin-pretreated SPF mice. (**C and D**) The Δ*SL1344*_*3500* mutant is not attenuated at systemic sites following intraperitoneal (IP) infection. Streptomycin-pretreated SPF mice were infected with 10^3^ CFU by IP injection (**C and D**) or by oral gavage with 10^7^ CFU (**D**) of the indicated tagged strains. Organs (liver, spleen, and mesenteric lymph nodes [mLN]) collected on day 4 post-infection were enumerated using differential plating. (**E and F**) Misexpression of *op2* suppresses growth in the gut lumen. Streptomycin-pretreated SPF C57BL/6 mice orally infected with an equal mixture of the indicated tagged strains at a total inoculum size of 5 × 10^7^ CFU. (**A–F**) The tested strains carried a chromosomal DNA tag, allowing their identification and quantification by qPCR for competitive index (CI) determination. CFUs for each strain were calculated based on their relative abundance, as determined by qPCR, and from the total *Salmonella* Typhimurium load in feces. Wilcoxon matched-pairs *≤0.05, **≤ 0.005.

The fitness defect of Δ*SL1344_3500* mutant was originally observed as a hit in RB-TnSeq screen performed in C57BL/6J mice colonized with a low complexity microbiota (LCM) (8) and was recapitulated in Fig. 3A. This mouse line has intermediate colonization resistance but is susceptible to *S*. Tm and permits reproducible colonization (22). In addition, the gnotobiotic OligoMM^12^ model was used, containing 12 microbiota strains (23). We observed no significant difference in fitness attenuation of Δ*SL1344_3500* in the context of different mouse microbiota models (Fig. 3A). In all mouse models tested, the mutant growth is progressively attenuated relative to WT, reaching 100- to 1,000-fold attenuation by day 4 post-infection (p.i.). Complementation of the deletion suggests that the fitness defect is specific to *SL1344-3500* (Fig. 3B).

The data imply that SL1344_3500 is important for gut luminal growth. To determine whether it also has a role in the dissemination to systemic sites, we used intraperitoneal (IP) injection to circumvent the initial phase of establishment in the gut. In a competitive infection by IP injection with the Δ*SL1344_3500* mutant and a congenic WT strain, we observed that about equal numbers of each strain were present at systemic sites (Fig. 3C). Once in the gut mucosa, *Salmonella* typically re-seeds into the gut lumen after 3–4 days of infection. We also found that approximately equal numbers of each strain reseeded the gut lumen at day 4 of the IP infection, whereas in the oral infection, there were significantly fewer of the mutant than the WT (Fig. 3D). In conclusion, SL1344_3500 deficiency is highly detrimental to gut luminal growth; however, it does not affect proliferation at systemic sites.

### Fitness burden is associated with the unregulated expression of operons 1 and 2

The *in vitro* and *in vivo* expression data indicate that SL1344_3500 represses the expression of the neighboring operons. While the expression of these operons may also be subject to regulation from other factors like catabolite repression or general stress response, the absence of SL1344_3500 markedly enhances expression of these operons and confers a fitness disadvantage during competitive infections. If the absence of repression attenuates the *in vivo* growth of the Δ*SL1344_3500* mutant, deleting operons 1 and 2 should alleviate the fitness burden. We constructed deletion mutants and assessed their fitness against the WT strain in an oral infection model. Deleting the entire region (Δ*op1* Δ*op2* Δ*SL1344_3500*) rescued the growth attenuation of the Δ*SL1344_3500* mutation (Fig. 3E). These observations suggest that the de-repression of *op1* or *op2* or both operons is causing the fitness attenuation of the Δ*SL1344_3500* mutant in the murine gut. As the genes in *op1* and *op2* may belong to different pathways, we aim to determine which operon imposes a fitness burden in the absence of SL1344_3500 repression. We generated *op1* and *op2* mutants in the background of Δ*SL1344_3500* mutant. The Δ*op1* deletion does not significantly improve the fitness of Δ*SL1344_3500* as demonstrated by the comparison of the Δ*op1* Δ*SL1344_3500* double mutant to the Δ*SL1344_3500* single mutant (Fig. 3F). In contrast, it is observed that the fitness of the Δ*op2* Δ*SL1344_3500* double mutant is notably greater than that of the Δ*SL1344_3500* single mutant, implicating that *op2* carries a fitness burden when *SL1344_3500* is deleted (Fig. 3F). Interestingly, deletion of *op2* alone results in a slight fitness disadvantage, indicating that *op2* supports growth but requires regulation (Fig. 3F). Our data provide genetic evidence that the absence of *op2* repression creates a fitness burden for *S*. Tm colonization in the murine gut.

### Plasmid stabilizing effect of SL1344_3500 gene complementation

**Fig 4:**
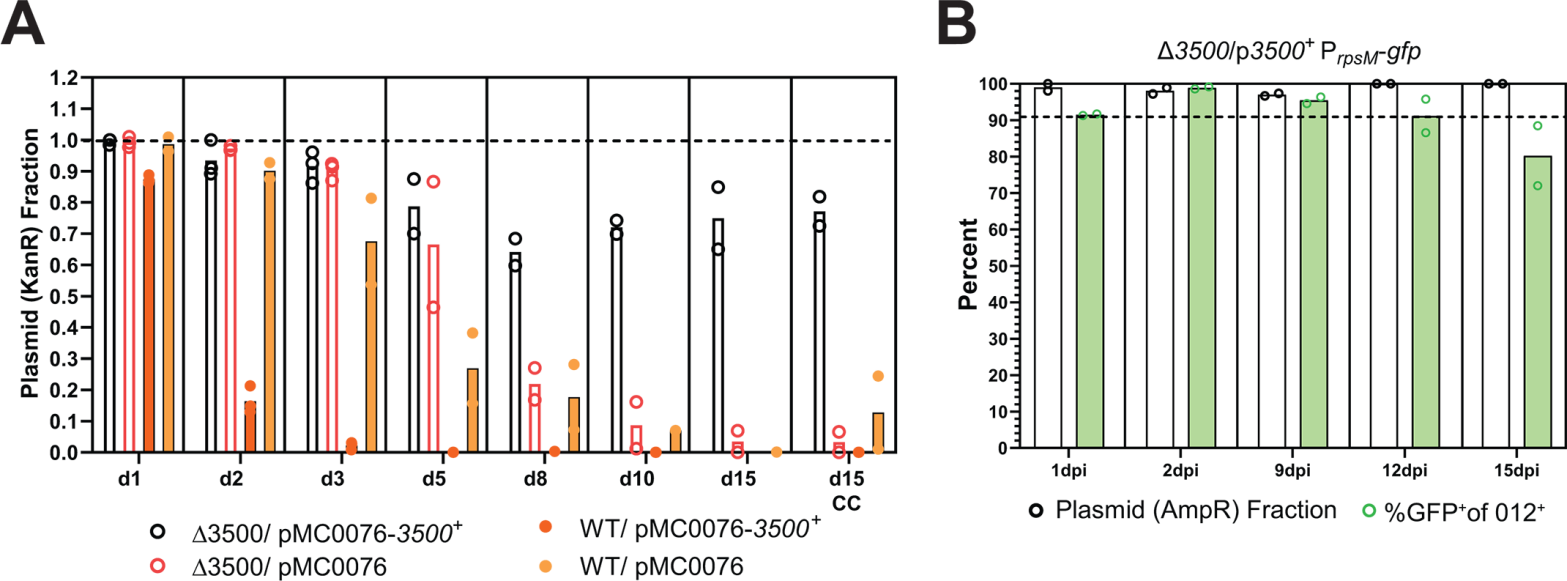
SL1344_3500 complementation enhances plasmid stability. (**A**) The *SL1344_3500* insert was cloned onto the pBR322 *oriP*-based vector, pMC0076, to construct pMC0076-3500^+^. Both plasmids carry a kanamycin resistance and were tested in the WT and Δ*SL1344_3500* backgrounds in oral infection of streptomycin pretreated 129S6/SvEvTac mice. Fecal samples were collected at specified times, and the fraction of *S*. Tm carrying the plasmid was determined by replica plating of the original CFU enumeration plate on plates with kanamycin. (**B**) 129S6/SvEvTac mice were infected with the Δ*SL1344*_*3500* mutant (Δ*3500*) strain that carries a hyperstable, constitutive GFP reporter (p*3500*^+^ *P_rpsM_-gfp*). This reporter contains a GFP gene driven by the *rpsM* promoter (*P_rpsM_-gfp*), a pSC101 oriP, and a copy of *SL1344_3500*. Fecal samples were collected at the indicated time points for plasmid retention analysis via replica plating and GFP expression analysis using flow cytometry. *S*. Tm was detected using O12 antisera, and GFP signals were analyzed within the O12-positive population.

Maintaining plasmid stability poses a challenge in long-term studies. While plasmids can be retained using antibiotics, this approach also disrupts the microbiota and interferes with experiments requiring an intact microbial community. Gene complementation may offer a strategy for improving plasmid maintenance, as positive selection of the complemented gene can offset the negative selection effects or intrinsic instability of the plasmid. Indeed, *SL1344_3500* gene complementation can stabilize the pMC0076 plasmid, which uses the widely used pBR322 origin. The pMC0076 plasmid demonstrated a burden of replication, as it was completely lost by day 3 p.i. in orally infected 129S6/SvEvTac (SPF) mice (Fig. 4A). In contrast, the plasmid carrying the complementation gene (pMC0076-3500^+^) in the Δ*SL1344_3500* mutant persists for over 15 days p.i. with plasmid retention of ~80% compared to around 1% for the backbone plasmid.

Plasmid loss *in vivo* has been observed in GFP reporter plasmids, purportedly due to high GFP expression that can suppress *S*. Tm growth. Plasmid loss was evident in our transcriptional reporter studies, even though the plasmid used has a stable *oriP* derived from pSC101 (Fig. 2B). Given the stabilizing effect of gene complementation, we constructed a hyperstable, constitutive GFP reporter plasmid incorporating the stable pSC101oriP and the *SL1344_3500* gene. This plasmid achieved nearly 100% retention and a high percentage of GFP^+^ cells for over 15 days of infection in the Δ*SL1344_3500* mutant (Fig. 4B). These findings demonstrate that complementing chromosomal deletion outweighs the replicative burden associated with propagating the plasmid. With this approach, it is possible to stabilize and ensure maintenance of functional plasmids even in long-term experiments without disrupting the ecosystem with antibiotic treatment. This grants an opportunity to equip *S*. Tm with functional plasmids that can provide new insights into the *S*. Tm lifestyle, metabolism, and interactions with the gut microbiota within the murine host.

## DISCUSSION

A previous transposon mutagenesis screen identified the gene *SL1344_3500* to be important for *S*. Tm fitness during infection of LCM mice (8). *SL1344_3500* encodes a putative IclR-like regulator, a family of regulators usually found to regulate metabolic operons, and here, we present data that further characterizes the role of *SL1344_3500* in *S*. Tm fitness.

We found *SL1344_3500* to be important during gut-luminal growth in several murine models (Fig. 3C and D). Interestingly, a small-scale genome association analysis showed that *SL1344_3500* homologs display a pattern of co-occurrence with two hypothetical operons. Homologs of *SL1344_3500* and the two operons (*op1*, *SL1344_3494-3496; op2*, *SL1344_3497-3499*) were predominantly found in non-typhoidal Clade A *Salmonella* serovars, which exhibit broader host tropism, and in human-adapted Paratyphi strains. However, they are nearly absent in Typhi strains and are rarely found in other host-adapted subspecies (Fig. 1A). This indicates that this genetic region is important for the ability to infect multiple host niches but could also indicate the process of negative selection against these genes, since host adaptation is an evolutionary young feature in *Salmonella* ssp.

The work presented here conclusively identifies SL1344_3500 as a transcriptional repressor of *op1* and *op2* expression. In the murine host, expression of *op1* and *op2* is normally repressed, but deletion of *SL1344_3500* increases expression from these operons (Fig. 2). Based on epistatic analysis, misexpression of *op2* is the main contributor to the fitness attenuation observed in Δ*SL1344_*3500, whereas *op1* appears to contain remnants of a transporter complex.

We provided evidence that *op2* encodes for xylonic acid utilization pathway. Transcriptional studies reveal that *op2* in the SL1344_3500-deficient strain is highly induced during stationary phase in complex medium, suggesting its induction might be driven by stress responses such as carbon limitation and hints at a role in carbohydrate utilization. Bioinformatics analyzes suggest that the *SL1344_3500* regulon may have arisen through gene duplication, as it shares a similar gene structure and encodes enzymes with high identity to the IclR-like YfaX and YjhI regulons in *E. coli* MG1655, which regulate the metabolism of the pentose acids rhamnoate and xylonate. (Fig. 1C). We further demonstrate that *S*. Tm can utilize xylonate as a sole carbon source, whereas the Δ*op2* strain cannot (Fig. S2). Given its role in xylonate metabolism, we propose designating the *op2* locus as *xynABC* and *SL1344_3500* as *xynR*.

Sugars can be oxidized by reactive nitrogen species, which are generated as part of host-mediated oxidative stress induced by antibiotics (24) and inflammation (25) to form sugar acids. The temporal availability of these sugar acids aligns with *op2*’s contribution to growth when inflammation is pronounced (days 3–4 p.i.; Fig. 3F), suggesting that xylonate or related metabolite may serve as a carbon source under these conditions. Gene fitness data from livestock animal models indicate that *op2* may be expressed and play a role in gut colonization in swine, cattle, and chickens (26). As livestock animals are a major reservoir for non-typhoidal *Salmonellae* in developed countries, it is of interest to further explore how the gut metabolite profiles of these hosts differ from those of murine models. Differences in sugar acid availability or microbiota composition may drive host-specific regulation of *op2*.

The uncontrolled expression of *op2* in the SL1344_3500-deficient strain leads to growth attenuation (Fig. 3F). This likely results from the accumulation of toxic metabolic intermediates due to imbalances in redox equivalents, carbon flux, and/or insufficient downstream enzymatic capacity. Intermediates of xylonate and rhamnoate catabolism include aldehydes (27), which can be toxic if accumulated in the cell (28–30). Such accumulation may occur when redox equivalents (e.g., NAD^+^/NADP^+^) are limiting, preventing complete oxidation. Since xylonate metabolism involves multiple oxidative steps (31), its efficient processing depends on sufficient redox balancing, which is compromised in the gut’s reducing environment due to a scarcity of suitable electron acceptors (32, 33). Only upon the onset of inflammation, when the gut environment shifts to a more oxidizing state (34–36), does *op2* expression positively impact growth (days 3–4 p.i.; Fig. 3F). Therefore, the growth defect observed with dysregulated *op2* expression likely stems from metabolic imbalance and the toxic effects of accumulating intermediates under low redox conditions, underscoring the importance of tight regulatory control over sugar acid catabolic pathways. This is consistent with the broader principle that incomplete carbohydrate metabolism leads to toxic intermediate accumulation, as exemplified by sugar phosphate toxicity resulting from disrupted metabolism of various sugars (37–39). These studies showed that the buildup of phosphorylated sugars or another intermediate, due to defects in downstream catabolic enzymes, leads to growth attenuation. Future work will aim to confirm the underlying molecular mechanism, as well as to assess the potential of targeting SL1344_3500 as an antimicrobial strategy. Its restriction to a limited set of species makes SL1344_3500 an attractive target for selectively inhibiting *Salmonella* without affecting the host microbiota.

Lastly, we were able to turn the fitness attenuation of the Δ*SL1344_3500* strain into a molecular tool to ensure reliable plasmid maintenance. Complementation of Δ*SL1344_3500* with plasmid expression resulted in increased stability of the plasmid by providing a selective advantage to maintain the plasmid (Fig. 4). While we have not presented a detailed study on the stability of various plasmid types, we are currently developing robust reporters with reliable GFP detection using stable oriP and *SL1344_3500* complementation. This opens the possibility to use plasmid-based assays to study important aspects of *S*. Tm pathogenesis and lifestyle during long-term infections.

## MATERIALS AND METHODS

### Construction of bacterial strains and plasmids

#### Bacterial strains

All *Salmonella* strains were constructed in *Salmonella enterica* serovar Typhimurium (*S*. Tm) strain SB300, which is a re-isolate of SL1344 (40) from a systemic mouse infection in the Galán lab (41).

Single-gene deletion strains were constructed using the λ Red recombination system by electroporating PCR products into WT *S.* Tm carrying the pKD46 plasmid, followed by selection of recombinant strains on lysogeny broth (LB) agar containing appropriate antibiotics (42). PCR products were generated from a pKD3 or pKD4 plasmid template with primers that contained 40 sequences of the gene-flanking regions. P22 phage lysates (43) were prepared from the mutant strains and used to subsequently transduce the mutations into the ancestral strain or a barcoded SB300 derivative. All strains are listed in Table S1.

#### Plasmids

The p3500^+^ plasmid was constructed by blunt-end cloning of the SL1344_3500 insert into the backbone of pDcuB (8). The backbone vector fragment, containing only the origin of replication (oriP) and the ampicillin resistance gene, was PCR-amplified from the pDcuB template using 5′ dephosphorylated primers (pDcuBcom5vF; PSC101ori_F). The insert was amplified from SL1344 genomic DNA using 5′ phosphorylated primers (SL1344_3500 + 43R; SL1344_3500-177F). The resulting amplicons were purified from isolated bands from gel electrophoresis and ligated using T4 DNA ligase. The pZ7907 plasmid was constructed by Gibson Assembly as follows: the Insert PsicA-gfpmut2 from pM974 was amplified by primers TBA84 and TBA85 to produce overhangs complementary to PCR linearized pZ7903 (empty pSC101 vector). PsicA was later excised by XbaI/PstI digestion and promoters of putative operon 1 and 2 were cloned by using Gibson Assembly to generate pZ7917 (P_op1_-gfp) and pZ7918 (P_op2_-gfp), respectively. Promoters of *op1* and *op2* were amplified from genomic DNA with primers that introduce overhangs to the digested pZ7907 (TBA110 and TBA111; TBA112 and TBA113). The pZ7919 plasmid (p3500^+^-P_rpsM_-gfp) was obtained by cloning P_rpsM_-gfp fragment (derived from pM965) into P3500^+^ plasmid by *XbaI-PstI* digestion and subsequent ligation. The pZ7904 plasmid (pMC0076-3500^+^) was constructed using Gibson Assembly of pMC0076 and the SL1344_3500 insert. pMC0076 was linearized by PCR amplification using primers TBA70 and TBA71. Insert SL1344_3500 was amplified by PCR to introduce overhangs to the backbone with primers, TBA72 and TBA73.

All constructed plasmids were transformed into cloning strain DH5α by electroporation, the insert sequenced, and lastly, transformed into *S*. Tm strains by electroporation. Plasmid and primers are listed in Table S1.

### Growth assay on xylonate

*S*. Tm strains were grown overnight in LB medium and then diluted to an OD_₆₀₀_ of 0.0001 (~1 × 10⁵ CFU/mL) in M9 minimal medium (42.3  mM KH₂PO₄, 64.3  mM Na₂HPO₄, 18.7  mM NH₄Cl, 8.6  mM NaCl, 1  mM MgSO₄, and 0.1  mM CaCl₂) supplemented with 0.5% casamino acids, trace elements (1  µM ZnSO₄, 5  µM MnSO₄, 1  µM CoCl₂, and 0.5  µM CuCl₂), 60  µM FeCl₃, and the indicated concentrations of lithium xylonate. Cultures were incubated at 37°C in microtiter plates under aerobic conditions with agitation.

### Mouse lines and mouse infection models

#### Mouse lines included

SPF C57BL/6J (Nramp^−/−^) and 129S6/SvEvTac (Nramp^+/+^) mice, and gnotobiotic mice (LCM [44] and OligoMM12 [23], both are of C57BL/6J [Nramp^−/−^] background). Mice used in the study were 8–12 weeks old and of either sex. SPF mice were originally obtained from Jackson Laboratories and subsequently bred in-house. Gnotobiotic mice were bred in flexible film isolators under strict exclusion of microbial contamination at the EPIC mouse facility, ETH Zurich, Switzerland. All mice were maintained on standard mouse chow (Kliba Nafag 3537; autoclaved), containing 4.5% fat, 18.5% protein, approximately 50% carbohydrates, and 4.5% fiber by weight.

#### Infection

SPF mice were pretreated with a single dose of 25 mg streptomycin by oral gavage one day prior to infection, while gnotobiotic mice were untreated. *S*. Tm oral infection was performed as previously described (45). In brief, bacterial cultures were grown in LB medium supplemented with 0.3 M NaCl for 12 hours and then sub-cultured for 4 hours. The cultures were then washed in phosphate buffered saline (PBS) and used to prepare an inoculum of 5 × 10^7^ CFU, which was administered by oral gavage. Feces were collected on the indicated days, and organs (liver, spleen, and mesenteric lymph nodes) were collected at the termination of the experiment. Samples were homogenized using a TissueLyser II (Qiagen), subjected to serial dilution, and plated on MacConkey agar supplemented with 50 µg/mL of streptomycin for enumeration of pathogen burden. Plasmid retention was estimated by replica plating of original plates used for pathogen enumeration on to MacConkey agar plate with the appropriate antibiotic selection of the plasmid. For IP infections, 12-hour *S*. Tm cultures from LB + 0.3 M NaCl were washed and diluted to infect mice with 10^3^ CFU, as previously described (46).

### Detection of SL1344_3494-3500 homologs in other *Salmonella* spp.

If available, 50 genomes for selected *Salmonella* subsp. from NCBI data sets (47) were selected, excluding metagenome-assembled genomes and atypical assemblies. The identifiers and taxonomic classification of all genomes are provided in Table S1. To identify putative homologs of the IclR-like regulator SL1344_3500 and its two neighboring operons (SL1344_3494 – SL1344_3499) within the selected *Salmonella* subsp., a reciprocal best hit search was performed using MMseqs2 “easy-rbh” v14.7e284 (48) against predicted protein sequences obtained by Prodigal v2.6.3 (49). Hits with *e*-value > 1e-10 and alignments covering less than 50% of the longer sequence’s length were discarded. For genomes where putative homologs for either the IclR-like regulator or any of the two neighboring operons were identified, the genomic neighborhood was investigated. To this end, neighboring ORFs were annotated against the UniProtKB/ Swiss-Prot database (50) using MMseqs2 v14.7e284 (--start-sens 1 –sens-steps 3 -s 7 –max-accept 10000). Hits with *e*-value >1e-10 were discarded, and the best hit was kept. Mainly, the presence of the *glp* operon (*glpDEG*) and the *glg* operon (*glgBXCAP*) was investigated.

### Distribution of SL1344_3494-3500 homologs among Enterobacteriaceae members

To explore the presence of SL1344_3494-3500 homologs within the broader family of Enterobacteriaceae, we downloaded all representative species classified as such from GTDB r220 (51) (Table S1). Genomes were downloaded using NCBI data sets (47) and processed as described previously. Additionally, the SL1344_3500 sequence was also blasted against the BioCyc database (52) to identify putative homologs in other members of Enterobacteriaceae. Blasted sequences with >70% identity were retained to minimize misidentification of other IclR-like regulators. The neighboring genes of identified orthologs were analyzed to assess their homology to the genes *SL1344_3494-SL344_3499*.

### qPCR analysis of barcoded mutant strains

Genomic DNA was extracted from 4-hour fecal enrichment cultures grown in LB medium (50 µg/mL streptomycin) and used as templates for quantitative PCR. Reagents included: FastStart Universal SYBR Green Master Mix (Roche, Cat# 4385610), with primers (see Table S1) at a final concentration of 1 µM. Equipment used: QuantStudio 7 Flex instrument (Applied Biosystems). Each barcode was analyzed with tag-specific primers and run in duplicates. The qPCR program consisted of an initial denaturation step at 95°C for 14 min, followed by 40 cycles of denaturation at 94°C for 15 seconds, annealing at 61°C for 30 seconds, and extension at 72°C for 20 seconds. Cycle threshold (Ct) values were back-calculated to a standard curve, generated using genomic DNA extracted from a single barcoded strain, to determine the relative abundance of each barcode. The competitive index was estimated relative to the WT strain as previously described (8, 37).

### RT-qPCR for transcriptional analysis

#### RNA extraction

RNA was extracted using the Qiagen RNeasy Mini Kit from pelleted cells in indicated growth phases. The protocol was performed with minor alterations. In brief, pellets were lysed in RTL buffer with β-mercaptoethanol (10 uL/mL) while heating to 80°C for 10 min. Samples were washed in 70% ethanol and the lysate filtered with the provided spin columns. The filters were washed in RW1 buffer and then twice in RPE buffer. RNA was eluted in RNAse-free H2O and stored at −80°C.

#### cDNA synthesis

Complementary DNA (cDNA) was synthesized with the Qiagen RT2 HT First Strand cDNA Synthesis Kit (Qiagen, Cat#330411) according to instructions. In brief, 1 µg of RNA from each sample was incubated in GE2 buffer for 10 min at RT for gDNA elimination. The samples were then mixed with RT BC4 Mastermix and cDNA synthesized with the following program: 42°C for 15 min, 95°C for 5 min.

#### RT-qPCR

Primers were designed to amplify 150 bp of the first gene in each putative operon, starting from five to seven codons of the predicted transcriptional start site. *RpoD* was used as an endogenous reference. Reactions using FastStart Universal SYBR Green Master Mix (Roche, Cat# 4385610) and primers at final concentration of 1.5 µM were set up and run with the following program on a QuantStudio 7 Flex instrument (Applied Biosystems): 95°C for 10 min, then 40 cycles of 95°C for 15 seconds, 60°C for 45 seconds, and 72°C for 30 seconds, with finally a melting curve measurement from 50°C to 95°C with 0.05°C/sec.

#### RT-qPCR analysis

The 2^−ΔΔCT^ method was used to estimate relative mRNA levels relative to the WT sample in each condition with RpoD as endogenous reference.

### Flow cytometry of transcriptional reporter strains

Inocula and fecal samples were pelleted, resuspended, and fixed in 2% paraformaldehyde (PFA) for 20 min, and washed in PBS + 1% bovine serum albumin (BSA) (wt/vol). Fixed samples were stored in the dark at 4°C for up to 4 days without loss of signal. Samples were then first stained with human anti-O12 antiserum (hSta5) (53) for 40 min, followed by two washes and staining of the secondary antibody, AlexaFluor (R) 647 AffiniPure Goat Anti-human IgG (Lubio Science GmbH) for 40 min. Samples were then analyzed using Beckman Coulter CytoFlex S flow cytometer. The *S*. Tm gate was set using the FMO control (pooled from fecal samples without primary antibody staining), and the GFP gate was set using GFP^+^ and GFP^−^ *S*. Tm control samples. The data were analyzed using FlowJo V10 (Tree Star) for Windows.

## SUPPLEMENTAL MATERIAL

Fig. S1 and S2 jb.00054-25-s0001.pdf Fig. S1: SL1344_3494-SL1344_3500 homologs in other Enterobacteriaceae members. Fig. S2: The ΔSL1344_3497-3499 mutant is unable to utilize xylonate as a sole carbon source.

Table S1 jb.00054-25-s0002.xlsx List of genome accessions, strains, and primers.

ASM does not own the copyrights to Supplemental Material that may be linked to, or accessed through, an article. The authors have granted ASM a non-exclusive, world-wide license to publish the Supplemental Material files. Please contact the corresponding author directly for reuse.
