# Supplementary material for: Identification of gene targets regulated by the IclR-like regulator SL1344_3500 in Salmonella Typhimurium
Source: J Bacteriol. 2025 Aug 25;207(9):e00054-25. doi: 10.1128/jb.00054-25 (PMC12445098; doi:10.1128/jb.00054-25)

Supplemental Figure 1. SL1344_3494-SL1344_3500 homologues in other Enterobacteriaceae members.

Supplemental Figure 2. The Δ*SL1344_3497–3499* mutant is unable to utilize xylonate as a sole carbon source . Strains were grown in M9 minimal medium with xylonate at the indicated concentrations at 37 °C under ambient atmosphere. Optical density (OD_{600}) was measured after 8 hours, and the starting OD_{600} was subtracted to calculate the ΔOD_{600}.

Supplemental Table 1. List of genome accessions, strains, and primers.

Supplemental Figure 1

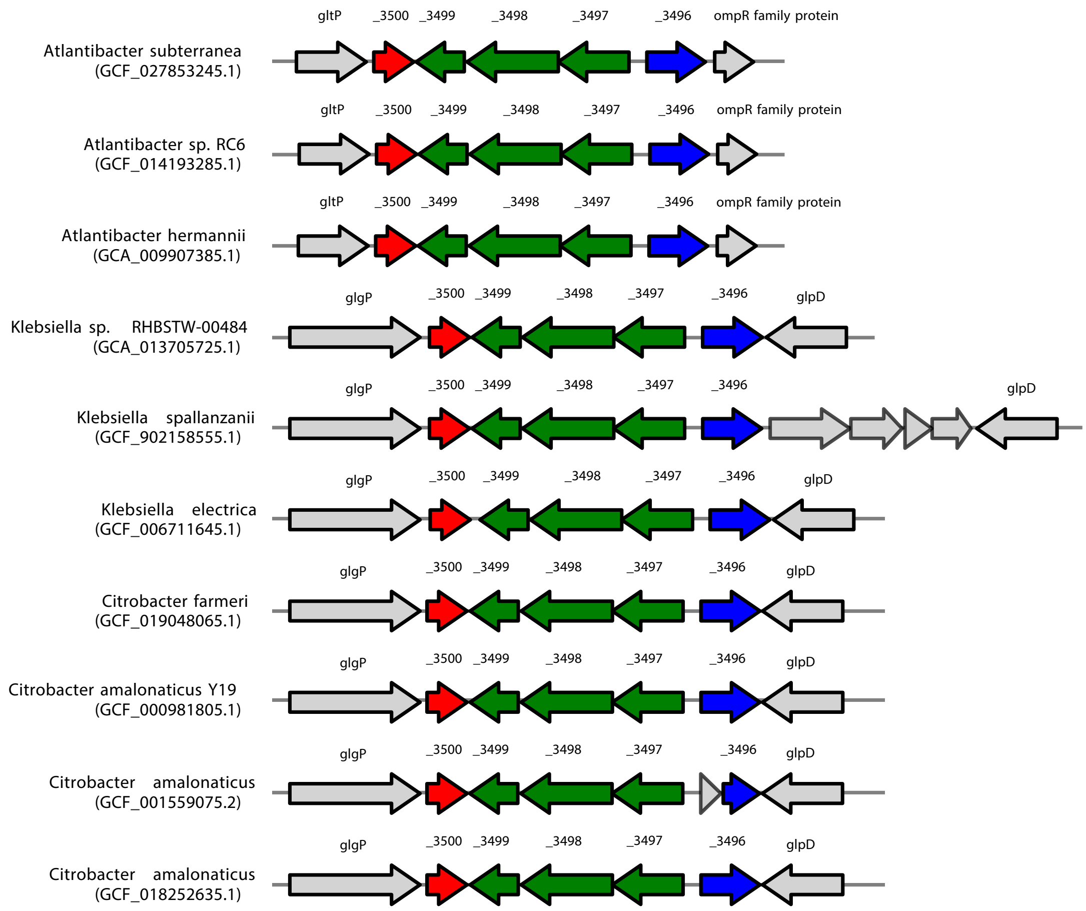

Supplemental Figure 2

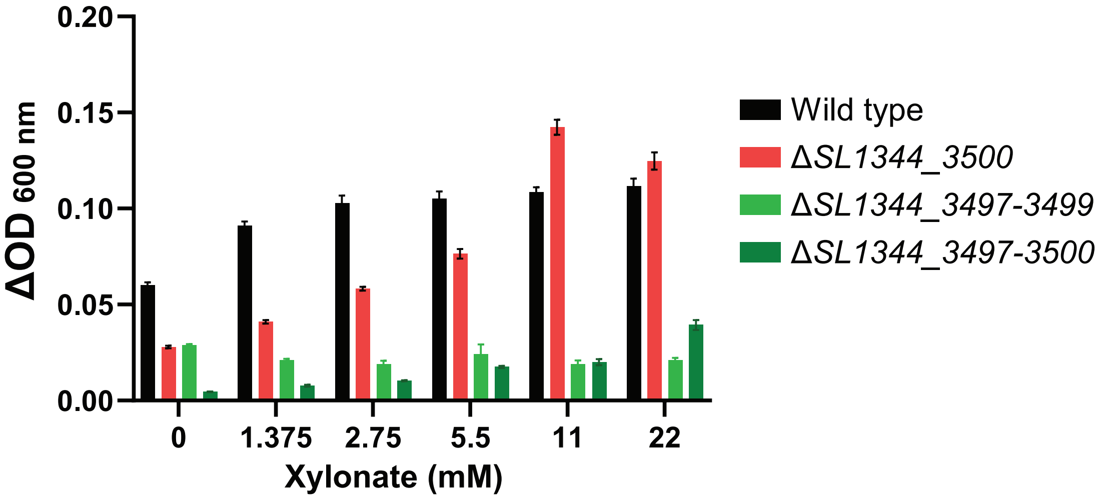

Supplement: Fig. S1 and S2 — Fig. S1: SL1344_3494-SL1344_3500 homologs in other Enterobacteriaceae members. Fig. S2: The ΔSL1344_3497-3499 mutant is unable to utilize xylonate as a sole carbon source. [file jb.00054-25-s0001.pdf]
